# Supplementary material for: Predicting visual function by interpreting a neuronal wiring diagram
Source: Nature. 2024 Oct 2;634(8032):113–23. doi: 10.1038/s41586-024-07953-5 (PMC11446822; doi:10.1038/s41586-024-07953-5)
Supplement: Supplementary file 6 — CRF and ERF predictions for individual TmY4 and TmY9 cells. Analogous to Supplementary Data 3, but for TmY target types. Shown are the top four monosynaptic pathways, the strongest pathway passing through each of the top ten intermediary types (ranking from Extended Data Fig. 7), and the trisynaptic pathway Tm1–TmY–Dm3–TmY (see the section entitled Prediction of spatial normalization). [file 41586_2024_7953_MOESM6_ESM.zip › DataS4/TmY9q/720575940627924501.pdf]

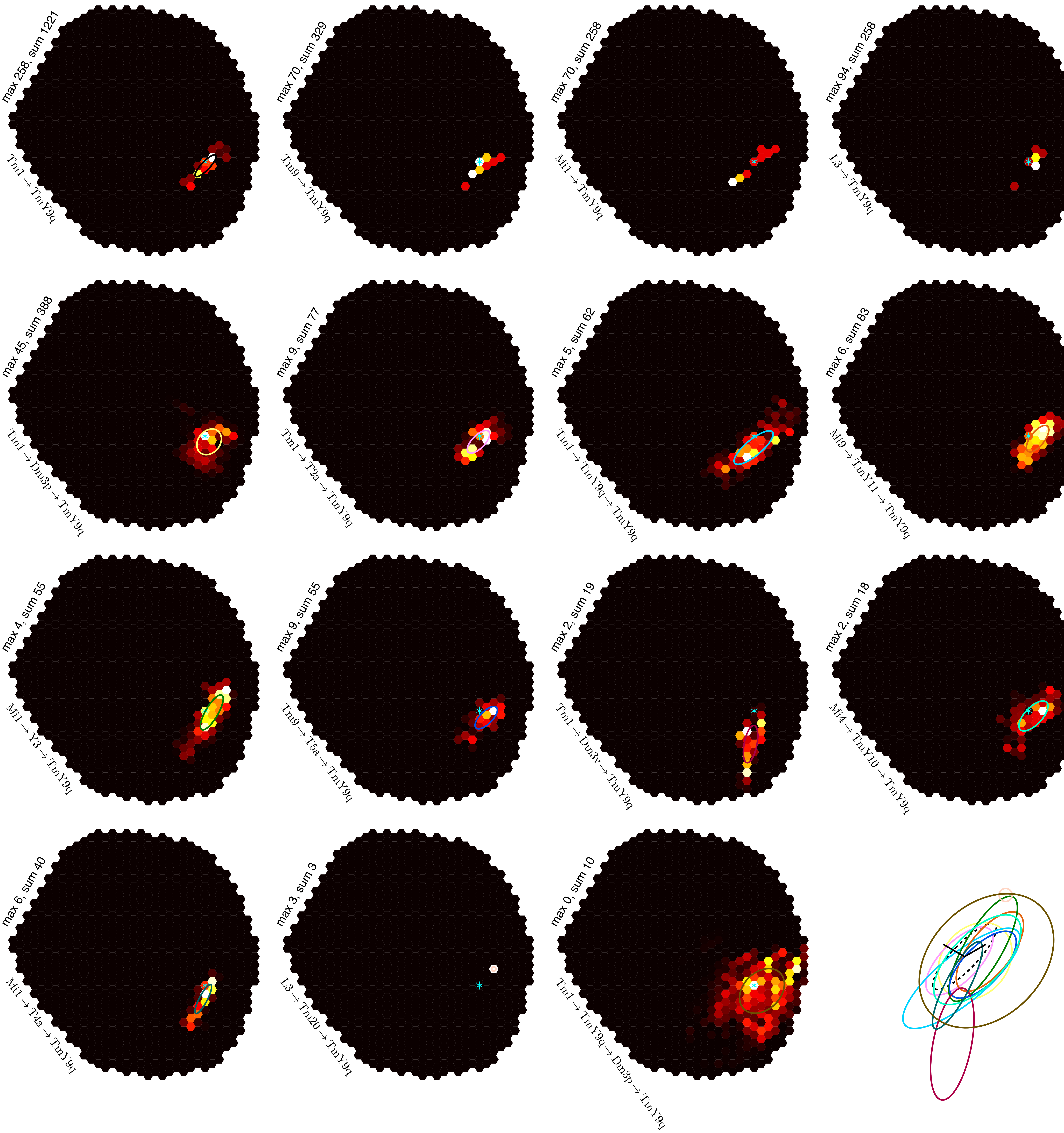
max 258, sum 1221
Tm1 → TmY9q
max 70, sum 329
Tm9 → TmY9q
max 70, sum 258
Mi1 → TmY9q
max 94, sum 258
L3 → TmY9q
max 45, sum 388
Tm1 → Dm3p → TmY9q
max 9, sum 77
Tm1 → T2a → TmY9q
max 5, sum 62
Tm1 → TmY9q → TmY9q
max 6, sum 83
Mi9 → TmY11 → TmY9q
max 4, sum 55
Mi1 → Y3 → TmY9q
max 9, sum 55
Tm9 → T5a → TmY9q
max 2, sum 19
Tm1 → Dm3v → TmY9q
max 2, sum 18
Mi4 → TmY10 → TmY9q
max 6, sum 40
Mi1 → T4a → TmY9q
max 3, sum 3
L3 → Tm20 → TmY9q
max 0, sum 10
Tm1 → TmY9q → Dm3p → TmY9q
